# Interaction of Swine Manure Ash-Oxygen Carrier Particles under Chemical Looping Conditions

**DOI:** 10.1021/acs.energyfuels.4c05071

**Published:** 2025-01-27

**Authors:** Iñaki Adánez-Rubio, Alberto Abad, Henrik Leion, Tobias Mattisson, Juan Adánez

**Affiliations:** † Instituto de Carboquímica (ICB-CSIC), Department of Energy & Environment, Miguel Luesma Castán 4, Zaragoza 50018, Spain; ‡ Energy and Materials, Chemistry and Chemical Engineering, Chalmers University of Technology, Kemigården 4, Gothenburg, SE 412 96, Sweden; § Department of Energy and Environment, Division of Energy Technology, Chalmers University of Technology, Göteborg, SE 412 96, Sweden

## Abstract

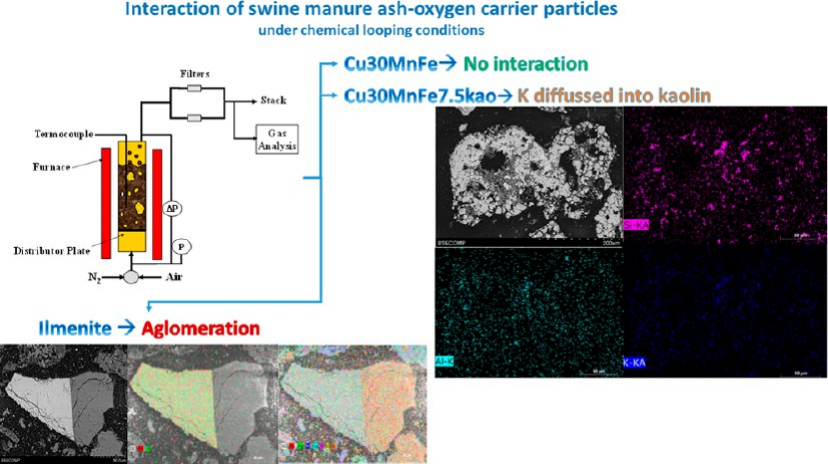

The interaction between biofuel ashes and the oxygen carrier in chemical looping combustion (CLC) and chemical looping with oxygen uncoupling (CLOU) processes will be a key factor for the future implementation of these processes on an industrial scale. This is important if the biofuel used is a waste product with a high ash content, as much as 30 wt %, as is dry swine manure. The main components of swine manure ash are Ca (17 wt %) and P (13 wt %). The present work studies the interactions between three different oxygen carriers, two synthetic magnetic Cu-based CLOU oxygen carriers (Cu30MnFekao7.5 and Cu30MnFe_Mag) and ilmenite, and swine manure ash. CLOU and CLC cycles were performed in a batch fluidized-bed reactor under harsh conditions using up to 33.3 wt % ash. For both CLOU oxygen carriers, the concentration of O_2_ released depended on rates of carrier conversion, although no agglomeration problems were found after 20 h of CLOU and CLC redox cycles with 25 wt % ash, and their CLOU reactivities also increased. However, the ilmenite sustained hard agglomeration after 20 h of CLC cycles with 25 wt % ash. After 20 h of CLC/CLOU redox cycles at 900 °C, all of the oxygen carriers showed ash particles adhering to their surface, with a higher degree of ash cover on Cu30MnFekao7.5 and ilmenite, both with minerals in their composition. Therefore, the presence of minerals in the oxygen carrier, either as a support or in the form of impurities (mainly Si and Al as kaolinite), could be related to a greater interaction with the ashes. Interaction with some ash elements resulted in ilmenite agglomeration, and the diffusion of K inside Cu30MnFekao7.5 particles was observed by using scanning electron microscopy coupled with energy dispersive X-ray (SEM-EDX), particularly on the kaolin-rich areas inside the oxygen carrier.

## 1 Introduction

The chemical looping combustion (CLC) process is a promising combustion technology for the capture of CO_2_ with a very low energy penalty.^1^ This technology was initially developed for the combustion of gaseous fuels and was adapted or modified for use with liquid and solid fuels.^1,2^ The use of solid fuels presents some advantages given their higher availability and versatility for energy generation. Moreover, the use of biomass or biowaste as fuel in the CLC process with solids fuels, coupled with carbon capture and storage (BECCS), is considered a negative emission technology (NET) as it reduces the CO_2_ concentration in the atmosphere.^3^

**Figure 1:**
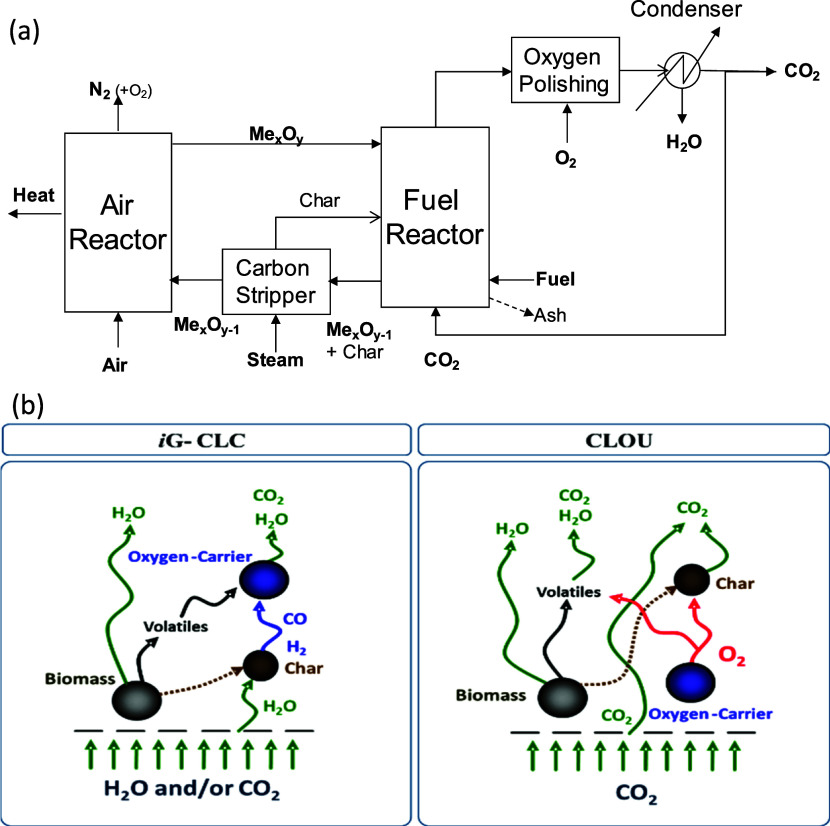
(a) Conceptual schematic diagram for the CLC process with solid fuels. (b) Reactions taking place in the fuel reactor during in situ gasification CLC (*i*G-CLC) and CLOU processes.

CLC technology involves combustion in two interconnected fluidized-bed reactors, the fuel reactor (FR) and air reactor (AR) (Figure 1a). A metal oxide, or oxygen carrier (OC), transports oxygen between the reactors. In this process, the fuel and N_2_ from the air are not mixed directly, meaning that it is not necessary to actively separate CO_2_ from the gas effluent, which reduces the energy penalty of the process.^1^

One option for the use of CLC technology with solid fuels is the process of chemical looping with oxygen uncoupling (CLOU). The main characteristic of the CLOU process is that the OC is able to release gaseous oxygen under the operating conditions inside the FR^4^ (see Figure 1b). This O_2_ gas burns the fuel as in conventional combustion, increasing combustion efficiency, to values reaching 100%.^2^ This is in contrast to conventional CLC, where the fuel reacts directly with the OC without releasing any O_2_.

One important aspect of CLOU is the interaction between the OC and the biofuel ash, which is rich in alkalis, such as Na and K, that can adversely affect the operation, causing problems of defluidization and agglomeration.^5^ Alkalis can also initiate the corrosion process and lead to fouling problems on the heat exchanger surface of the heat recovery system.^6^ Moreover, alkalis (K and Na) increase the gasification reactivity of chars from both coal and biomass.^7−9^ In fact, the catalytic effect of K impregnated on coal and biomass chars on gasification reactivity is well-known. In recent years, only limited studies have been conducted on the interaction of OCs, mainly ilmenite and Mn-based, with ash compounds in different CL processes with biomass at different scales: batch fluidized bed,^9,10^ CL continuous units for combustion^11,12^ and gasification,^13^ and oxygen carrier-aided combustion (OCAC) units.^12,14^ Focusing on batch studies, Lu et al. investigated the interaction between K and ilmenite in a fluidized-bed reactor for OCAC by injection of a K solution into one ilmenite bed.^15^ They found that K initially concentrated on the ilmenite surface before penetrating the particles as the operating time increased, and they found KTi_8_O_16_ to be the only crystalline compound. Moreover, K absorption did not affect ilmenite redox reactivity.^15^ Staničić et al. studied the interaction between individual ash compounds with synthesized Mn-based OCs, natural ores, and synthetic ilmenite.^9^ They found that OCs with Si in their composition had a greater tendency to agglomerate in the bed and that the presence of iron compounds decreased the agglomeration tendency even when this OC had a higher propensity to absorb alkalis from the studied materials.^9^ Natural ores were found to react more with the ash compounds compared to synthetic ilmenite, which is a pure Fe and Ti mixed oxide,^16^ resulting in particle agglomeration. This could be related to the presence of mineral impurities in the ores that preferentially react with the ash compounds. In addition, Purnomo et al. analyzed the effect of potassium salts, present in biomass ash, on different Fe-based OCs.^17^ They used potassium salts (K_2_SO_4_, KH_2_PO_4_, and K_2_CO_3_) mixed with the OCs in a crucible under a reducing atmosphere (2.5% H_2_ + 10% steam in Ar and N_2_) for 3 h at 900 °C. Using scanning electron microscopy coupled with energy dispersive X-ray (SEM-EDX), they found that the K from K_2_SO_4_ and K_2_CO_3_ diffused inside the particles, and that the K from the KH_2_PO_4_ formed an external layer over the particle.^17^ They also observed that the higher the amount of Si in the composition, such as the iron sand OCs with 16 wt % Si, the higher the tendency of the OC to agglomerate in the presence of K salts.^17^ This is due to the high affinity between K and Si, inducing the formation of potassium silicates with a low melting.

On the other hand, the K present in the biomass has been shown to increase gasification reactivity in CLC/gasification processes.^18^ Mei et al. analyzed the combustion by in situ gasification CLC (*iG*-CLC) of charcoals impregnated with carbonates (Na and K) and chlorides (Na and K) with a Mn-ore OC. They found that the presence of K and Na in the char improved the gasification rate of the charcoal by a factor of between 3 and 10, with improvement being more pronounced in the presence of carbonates than with chlorides.^10^ However, all of the impregnated charcoals caused the OC to experience agglomeration problems, which was more pronounced with K compound-impregnated charcoals.^10^

Moreover, different agglomeration-related behaviors were found. In the experiments performed with carbonates, Mei et al. found that the presence of K enhanced the melting of the OC surface that caused the agglomeration to occur, and in the case of Na compounds, they found areas high in Na–Si–Ca with a low melting point and conducive to agglomeration. The Si and Ca in the OC came from the mineral impurities present in the Mn-ore. On the other hand, with chloride compounds, the agglomeration bridges formed were high in K–Si–Ca and Na–Si–Ca in all cases.^10^

Most CLOU-related experience has been achieved using coal or biomass as fuel.^19−22^ Studying the interaction between one Cu-based OC for CLOU and coal ash, Dai et al.^22^ proposed two possible CuO/coal ash interactions, including the formation of a liquid phase at low temperature driven by y alkali species (Na and K), and the solid–solid reaction with Fe_2_O_3_ and Al_2_O_3_ to generate Cu–Fe and Cu–Al complexes with deactivation of the OC. A recent study by Filsouf et al. analyzed the interaction between ash and OCs in the CLOU process by burning pine sawdust biomass in a 1.5 kW_th_ CLOU continuous unit using two different Cu-based magnetic OCs.^23^ While Ca was the main element found in the biomass ash, it did not show any interaction with either OC, and both materials were easily separated from the main ashes, owing to their magnetic properties. On the other hand, they found that only the OC containing kaolin (Al_2_Si_2_O_5_(OH)_4_) in its composition interacted with the K and Mg from the biomass ashes and doubled their amount in the OC particles after 56 h of combustion.^23^ Moreover, they observed that although K diffused inside the particle in areas enriched with kaolin, this did not occur in areas of the active phase that were rich in Cu, Mn, and Fe.

In addition to the possibility of achieving pure CO_2_ in the fuel reactor outlet stream, CLC has several other aspects of great interest that could make this conversion technology highly applicable for waste and “dirty” fuels. The decoupling of the oxidation and fuel conversion, as seen in Figure 1, means that ash species could be concentrated in the fuel reactor, keeping the air reactor largely free of aggressive species. If released into the gas phase in the fuel reactor, impurities would be relatively concentrated and more easily removed. As most of the heat transfer surfaces would be present in the air reactor, low corrosion could be expected during CLC even when using fuels with high ash contents. Some works have shown the possibility of using waste materials as fuels for the CLC process, e.g., plastics^24^ and sewage sludge,^25^ showing lower environmental concerns than in a conventional process.

Agro livestock is a major economic activity in Europe. About one-third of farms are dedicated to rearing livestock for food, with pig farming accounting for 35%.^26^ Europe is currently the world’s second largest pig-farming region, and this activity largely involves intensive farming practices. Spain is the leading producer of pigs in Europe, and continued growth is expected in the country in the coming years, unlike in others such as Germany, France, and Italy.^27^ Spain has a pig population of 30.8 million head (second quarter of 2019), almost 50% of which are reared in the regions of Catalonia and Aragon, which produce about 62 million tons of swine manure per year.^26,27^ This situation makes the management of swine manure a critical issue for sustainability in the pig-farming sector. In fact, new legislation is restricting the expansion of pig farms in certain saturated zones unless there is an appropriate strategy in place for the treatment of swine manure.^28^

Swine manure is a waste product mainly consisting of a mixture of solid and liquid excrement as well as food scraps and cleaning water. Its composition can vary depending on very different factors such as the age of the pig, feeding practices, cleaning practices, type of storage, storage time, and weather conditions. However, it is generally described as a liquid effluent, or slurry, with a very high biochemical oxygen demand (BOD = 13,400–40,000 mg O_2_·L^–1^), high content in macronutrients, such as nitrogen (3000–5200 mg·L^–1^) and phosphorus (660–920 mg·L^–1^), and contaminated by some trace elements (Cu, Zn) and pathogens, such as fecal coliforms.^29^ What is more, these compounds contain a large amount of ash, typically 25–35 wt % on a dry basis, and are high in Na, K, and P. Because of these characteristics, inadequate management of this waste can lead to important environmental problems, such as contamination of soil and groundwater by N- and P-containing compounds, which cause the eutrophication of water, and emissions of nitrogen-containing compounds (NH_3_, N_2_O) and CH_4_ into the atmosphere, contributing to acidification and enhancing the greenhouse effect. Treatment by CLC or CLOU for heat and power could be a highly interesting opportunity for this type of fuel. Only one study has been found that deals with the conversion of swine manure by the CLOU process (with 30 wt % ash), which achieved as much as 99% CO_2_ capture efficiency at 950 °C and found that around 95% of the N from the fuel was converted to inert N_2_.^30^ For the use of these processes in the treatment of swine manure, it is necessary to know the effect of ash compounds on OC behavior.

The aim of this work is to study the interaction of OCs with swine manure ash and its effect on the OC reactivity and fluidization behavior. For this purpose, experiments were performed in a fluidized-bed reactor with three different OCs: two magnetic Cu-based OCs and ilmenite. The two Cu-based magnetic OCs (Cu30MnFekao7.5 and Cu30MnFe_Mag) were developed at the ICB-CSIC for the CLOU process. They showed good performance in the combustion of different fuels,^20,21^ such as different coals, biomass, and swine manure.^30^ Ilmenite is well-known for its importance in the CLC process with solid fuels^2^ and is used here for comparison with both CLOU OCs. The experiments studied O_2_ release in inert N_2_ (CLOU cycles) and conversion of CH_4_ (CLC cycles) in two series of interactions with swine manure ashes. The oxygen release capacity of the CLOU OCs was analyzed in relation to the amount of ash in the bed over time. CH_4_ combustion by the OCs over time was also analyzed. The used OCs were characterized by SEM after operations in the batch fluidized-bed reactor to observe the interaction between the OCs and the elements in the ash.

## 2 Experimental Section

### 2.1 Oxygen Carriers

Three different OCs were used in the present work: two synthetic Cu-based OCs and one ore as ilmenite. The synthetic OCs used were Cu30MnFe_Mag^20,31^ and Cu30MnFekao7.5,^21,32^ both developed and prepared by ICB-CSIC. Owing to their high Fe content, these materials are magnetic. Both OCs were prepared by using a high-shear mixer and granulator (Eirich laboratory mixer EL1). The granules were subsequently calcined at 1100 °C for 2 h (Cu30MnFekao7.5) or 4 h (Cu30MnFe_Mag). The raw materials used to prepare the kaolin-reinforced OCs were CuO (Panreac), Mn_3_O_4_ (Micromax, Elkem), Fe_2_O_3_ (Acros Organics), and kaolin (aluminosilicate, Sumitomo Seika). The calcined granules were sieved in the particle size range 0.1–0.3 mm for their use in the batch fluidized-bed reactor. The main phases of Cu30MnFe_Mag were Fe_2_O_3_, Fe_5_CuO_8_, and Cu_0.5_Mn_0.5_FeO_4_; while for Cu30MnFekao7.5, the main phases were Cu_0.5_MnFe_1.5_O_4_ and Cu_1.5_Mn_1.5_O_4_. Both OCs were prepared in a similar manner, and both have a triple oxide in their composition. However, they exhibited different main phase. The ilmenite^33^ was a concentrate from the mineral ore and was calcined at 950 °C for 24 h and activated. Table 1 shows the main characteristics of the three OCs used in the present work.

**Table 1:** Properties of Fresh OCs

	Cu30MnFe_Mag	Cu30MnFekao7.5	ilmenite
oxygen transport, *R*_OC,CLOU_ (wt %)	2.0	2.3	
oxygen transport, *R*_OC,CLC_ (wt %)	4.0	4.6	4.8
crushing strength (N)	2.2	1.8	2.2
skeletal density of particles (kg/m^3^)	5125	4720	4100
porosity (%)	51.6	29.5	1.2
specific surface area, BET (m^2^/g)	<0.5	<0.5	0.8
XRD main phases	Fe_2_O_3_, Fe_5_CuO_8_	Cu_0.5_MnFe_1.5_O_4_	Fe_2_TiO_5_,Fe_2_O_3_
	Cu_0.5_Mn_0.5_FeO_4_	Cu_1.5_Mn_1.5_O_4_	TiO_2_

### 2.2 Manure Ash

The ash used in this work was prepared from dry swine manure that was sourced from a closed-herd pig farm in northern Spain. The drying procedure is described elsewhere.^34^ The dry swine manure, with a particle size of 0.5–3.35 mm, was burned in a muffle furnace at 900 °C for 6 h to obtain the ash. With regard to the calcination conditions, to produce only swine manure ashes, a temperature of 900 °C was chosen as the typical operating temperature in the FR in CLC processes with solid fuels. However, the calcination time was selected after several tests in order to completely eliminate carbon and leave only ashes. The ash composition, determined by inductively coupled plasma-optical emission spectroscopy (ICP-OES), is given in Table 2. It shows the high amounts of Ca and P present in the manure, followed by Mg, Si, and smaller amounts of K, Fe, and Na. The large amounts of Mg and Si can be partially explained by the sepiolite used in pig farming as a liquid absorbent. The main phases, determined by X-ray diffraction (XRD), can also be seen in Table 2. The main P phases are Mg_2_P_2_O_7_ and Whitlockite (Ca_2.71_Mg_0.29_(PO_4_)_2_). The Ca is found in several phases, such as CaO, CaCO_3_, and CaSO_3_. The Si is found as SiO_2_.

**Table 2:** Swine Manure Ash Composition Obtained by ICP-OES Analysis and Main Phases Obtained by XRD

swine manure ash elements (wt %)			
Al	0.8	Mn	0.3
Ca	17	Na	1.1
Fe	1.1	P	13
K	1.8	Si	6.3
Mg	8.9	Ti	0.03

main phases			
Mg_2_P_2_O_7_, Ca_2.71_Mg_0.29_(PO_4_)_2_, Ca_10_(PO_4_)_6_S, CaSO_4_, SiO_2_, CaO, CaCO_3_			

### 2.3 Experimental Setup

**Figure 2:**
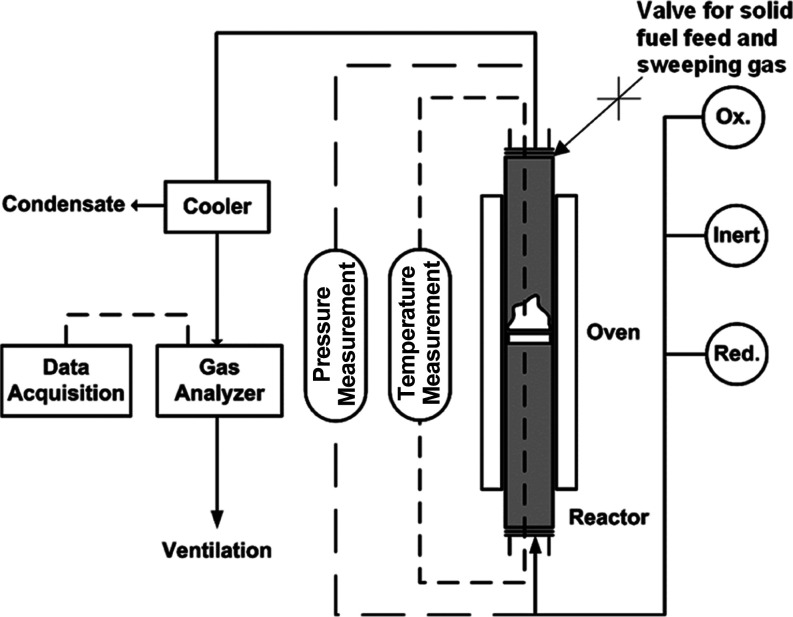
Schematic diagram of the experimental setup used in this investigation.

Experiments were performed in a quartz fluidized-bed reactor with a height of 870 mm and an inner diameter of 22 mm with ash addition. Figure 2 is a schematic diagram showing the experimental setup used in the present work. The OCs were placed on a porous quartz plate at a height of 370 mm from the bottom of the reactor. The reactor temperature was measured by two thermocouples located about 5 mm below and 25 mm above the plate (just above the OC bed), respectively. The pressure drop in the reactor was measured by a pressure transducer at a frequency of 20 Hz in order to analyze the fluidization behavior of the OC in the bed during the experiments. The exit gas stream from the reactor was led to a condenser to remove any water produced during CH_4_ combustion. The composition of the dry gas was measured by a Rosemount NGA-2000 analyzer as the concentration of O_2_ passing through a paramagnetic channel; CO_2_, CO, and CH_4_ passing through infrared channels; and H_2_ by thermal conductivity. More information about the experimental setup can be found elsewhere.^10^

### 2.4 Experimental Planning

Two different series of experiments were performed to analyze the interaction between the manure ash and the OCs: (1) Experiments with increasing ash load in the OC bed; (2) long-term experiments with a fixed fraction of ash in the bed. In both series, 15 g of OC was placed on the fluidized-bed reactor and it was heated to 900 °C in 11% O_2_-balance N_2_ mixture to fully oxidize the OC. From that point on, redox cycles would entail OC reduction in inert gas (N_2_) or fuel, followed by oxidation with the previously described 11% O_2_ mixture.

The series 1 experiments were performed with Cu30MnFekao7.5 and consisted of CLOU cycles of inert gas (300 s)-11 vol % O_2_ with a total flow of 600 mL/min, while increasing the amount of ash in the bed from 0 to 33.3 wt %. The ash was loaded with a pressurized feed system described elsewhere.^10^ Seven loads of different amounts of ash were added: 0.2, 0.2, 0.5, 0.5, 1.5, 2, and 2.5 g, with a total amount of 7.5 g of ash in the bed by the end of the series. Three CLOU cycles were run before the first load was added. Each ash load was added at the start of the inert period of a CLOU cycle, followed by three more CLOU cycles after the load was added. A total of four CLOU cycles were run with each ash load in the bed. A total of 31CLOU cycles were run in this series (see Table 3). This methodology of increasing the ash/OC ratio enabled the OC reactivity to be assessed under increasingly intense conditions. The series also studied the maximum allowable ash concentration in the bed that would not cause agglomeration problems for steady-state operation on an industrial scale. A similar procedure was utilized by Azis et al. with coal ashes, but only for testing under CLC conditions.^7^ After series 1 was conducted with Cu30MnFekao7.5, it was found to be of more interest to start with a fixed amount of ash in the bed and carry out several hours of redox cycles in order to maximize the contact between the OC and the manure ash.

**Table 3:** CLOU and CH_4_ Combustion Cycles were Run in Series 1 and 2

series 1								
ash load (wt %)	0	1.3	2.6	5.7	8.5	16.2	24.6	33
N_2_-air cycles	4	4	4	4	4	4	4	4
CH_4_ cycles	---	---	---	---	---	---	---	---
total cycles	3	7	11	15	19	23	27	31
series 2								
ash load (wt %)	25	25	25	25	25	25	25	25
N_2_-air cycles	3	3	3	3	3	3	3	3
CH_4_ cycles	4	4	4	4	4	4	4	4
total cycles	7	14	21	28	35	42	49	56

All 3 oxygen carriers were used in series 2. For this purpose, a load of 25 wt % ash (5 g) was mixed with 15 g of OC, and three CLOU cycles were run out at 900 °C (300 s) to study oxygen release with the ash. Subsequent to the O_2_ release cycles, fuel cycles were run with CH_4_ in pulses of 5 or 10 s with 100% CH_4_ at a flow rate of 450 mL/min. These CH_4_ pulses kept OC conversion at between 15 and 30%, simulating oxygen-to-fuel ratio values of between 6.7 and 3.3. Nitrogen was used as an inert purge for 120 s between oxidation and reduction. After 5 h of pulses, three CLOU cycles were run to analyze the effect of the ash-OC interaction during oxygen release. As ilmenite does not have CLOU properties, CLC redox cycles with CH_4_ were carried out with this OC to analyze its interactions with the ash. CLC/CLOU cycles were run for a total of 20 h with each oxygen carrier. CH_4_ was chosen as a model fuel gas compound to evaluate the OC reactivity and possible changes occurring to it. Significant fractions of this compound can also be expected to be present when swine manure is used, with a large volatile fraction expected in the bed.^35^

### 2.5 Data Analysis

eq 1 was used to calculate reduction conversion *X*_red_ as a function of time during the reduction period from the measured concentrations of different gaseous species in the gas analyzer:
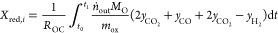
1where *X*_red,*i*_ is the instantaneous mass-based conversion at time *t*_1_, *n*_out_ the molar flow rate of dry gas at the reactor outlet as measured by the analyzer, *M*_O_ the molar mass of oxygen, and *t*_0_ and *t*_1_ the initial and final time of measurement.

The reactivity of a given oxygen carrier is quantified in terms of CO_2_ yield, γ, and is defined as the fraction of fully oxidized fuel divided by the carbon containing gases in outlet stream, in this work CO_2_, CO, and CH_4_.
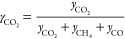
2

Here, *y*_*i*_ denotes the concentration (vol %) of the respective gas, obtained from the gas analyzer.

## 3 Results

### 3.1 Effect of Ash Addition in CLOU Experiments

**Figure 3:**
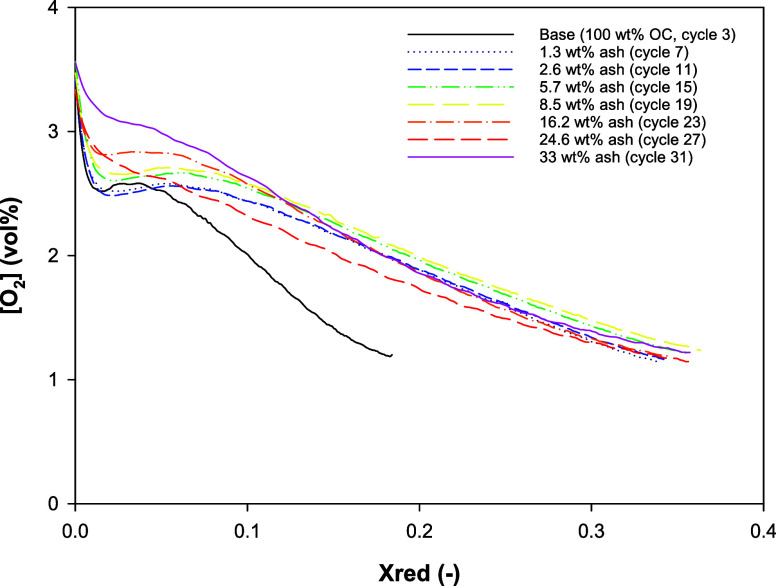
Oxygen concentration as a function of OC conversion for different ash loads. Series 1. Cu30MnFekao7.5. 900 °C. Reduction: N_2_; oxidation: 11 vol % O_2_.

The first series of experiments was performed to analyze the effect of the ash load on the oxygen release behavior using OC Cu30MnFekao7.5. Figure 3 shows the oxygen concentration released by the OC over every four cycles with different ash loads as a function of reduction conversion. The O_2_ concentration decreased with the degree of solid conversion, exhibiting the typical behavior found with mixed oxide OCs with and without kaolin.^36^ Moreover, the OC showed an increase in the oxygen released during the first 4 CLOU cycles, with an ash load of between 0 and 1.3 wt %. This activation was also found in previous studies with Cu-based magnetic OCs,^31,32^ where their oxygen transport capacity increased with the cycles in a batch fluidized-bed reactor.

Furthermore, the shape of the curve changed for ash loads higher than 16.2 wt %. With ash loads between 0 and 16.2 wt %, there was a period where the release of the O_2_ did not depend on conversion and was stable from reduction conversion of 0 to 0.1. Once the conversion reached 0.1, the O_2_ concentration started to decrease with conversion. However, with loads higher than 16.2 wt %, the O_2_ concentration decreased over time from the beginning of the reaction. This change in the O_2_ release behavior could be a result of the interaction between the OC and the ash. The mechanism by which the oxygen release behavior changes when there is a high concentration of ash in the bed is unknown. No changes were detected in the XRD phases present in the particles used (XRD analyses are not given because they do not show a significant result). Thus, the mixed oxides that govern the O_2_ releases by the OC did not undergo any change that would modify the thermodynamics of the process. The only change detected in Cu30MnFekao7.5 was an increase in K in the kaolin phase (Sections 3.3.1 and 3.3.2), although kaolin does not have CLOU properties. Therefore, more research in this area is required.

After 31 CLOU cycles, the OC extracted from the batch fluidized-bed reactor was well mixed with the ash and did not suffer from any agglomeration. Moreover, the OC sustained and even increased its reactivity from the first few cycles, notwithstanding a high ash load of 33.3 wt %, without any agglomeration problems.

### 3.2 Effect of Ash-OC Contact Time in CLC/CLOU Experiments

#### 3.2.1 Oxygen Release Behavior by CLOU OCs

**Figure 4:**
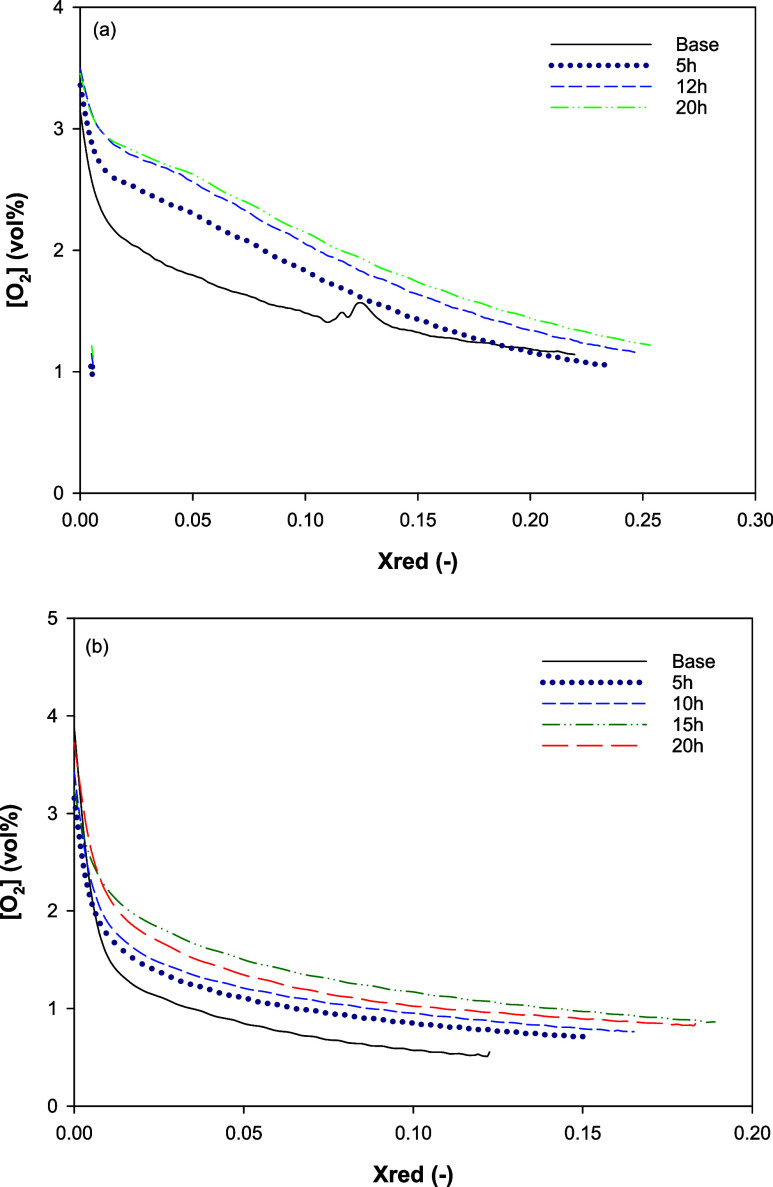
Oxygen concentration as a function of OC conversion with the operating time. Series 2. (a) Cu30MnFekao7.5; (b) Cu30MnFe_Mag. 900 °C. Reduction: N_2_; Oxidation: 11 vol % O_2_.

Series 2 studied the effect of interaction time between the OCs and the manure ash (25 wt % of the bed). To enable comparison between the behavior of ilmenite (without CLOU properties) and that of Cu-based CLOU oxygen carriers, CH_4_ was used as fuel during pulses of 5 or 10 s with all of the OCs in order to reach maximum OC conversions of between 15 and 30%. In the case of the CLOU OCs, several CLOU cycles were run every 5 h of operation to analyze the release of the O_2_ release. Figure 4 shows the oxygen concentration released by OCs Cu30MnFekao7.5 (Figure 4a) and Cu30MnFe_Mag (Figure 4b). The oxygen released by the OC increased over time, as had been previously observed with these OCs without any ash interactions.^31,32^ The increase in the O_2_ concentration was much lower at high reaction times, an effect that was more pronounced for the Cu30MnFekao7.5. Higher released O_2_ concentrations were found in all cases for the materials containing kaolin.

In contrast to the observations made in series 1 for Cu30MnFekao7.5 with 25 wt % ash, the released oxygen concentration directly depended on the OC conversion in every cycle. Therefore, this change in the oxygen release behavior could be associated with the interaction between the manure ash and the oxygen carrier. With respect to Cu30MnFe_Mag, the effect of the ash was negligible compared to the results obtained in previous works by the group during the development of this oxygen carrier.^31^Figure 4b shows the same oxygen release behavior as that in experiments in the fluidized-bed reactor without ash. In both cases, after 20 h of redox cycles, the oxygen carriers extracted from the batch fluidized-bed reactor were well mixed with the ash and did not experience agglomeration. Moreover, the interaction with ash did not reduce the OC reactivity for CLOU.

#### 3.2.2 CH_4_ Combustion with Ilmenite

**Figure 5:**
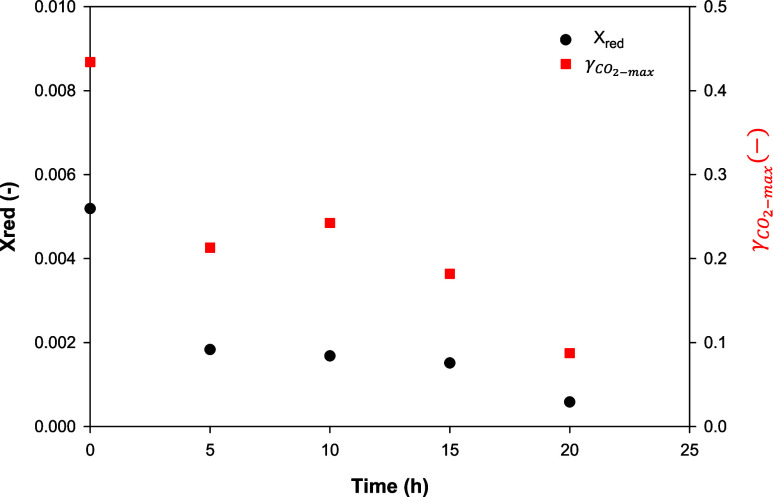
CO_2_ and reduction conversion as a function of the operating time with ilmenite. Series 2. 900 °C. Purge: N_2_; Reduction: 5s CH_4_; Oxidation: 11 vol % O_2_.

With regard to the CLC cycles run using ilmenite as the OC, Figure 5 shows the maximum CO_2_ yield and the OC reduction conversion as a function of operating time, operating under the same conditions as in previous experiments. It can be seen that from the beginning the ilmenite did not convert the CH_4_ to a high extent, given the maximum γ_CO_2__ values of about 0.42, which had previously been observed in the literature.^33^ The CO_2_ yield decreased during the first 5 h, reaching a maximum value of 0.25. This behavior was sustained over the following 15 h. Moreover, at the same time, there was a small decrease in the pressure drop of the bed, although it continued to fluidize. These details can be associated with a partial agglomeration of the bed.

However, after 20 h of redox cycles, the ilmenite in the bed agglomerated during reduction with CH_4_, decreasing the maximum CO_2_ yield to half that of previous experiments and resulting in an OC reduction of close to 0. During oxidation, fluidization was partially restored and a new redox cycle was carried out; as in the previous cycle, the bed agglomerated and was not restored during oxidation. Therefore, the ilmenite extracted from the batch fluidized-bed reactor after 20 h of redox cycles was completely agglomerated with the ash in the bed.

### 3.3 OC Characterization

After the experiments carried out in the batch fluidized-bed reactor, the OC particles extracted from the bed were analyzed by SEM using an ISI DS-130 scanning electron microscope coupled with an ultrathin window PGT Prism detector for EDX analysis.

#### 3.3.1 Ash Addition in CLOU Experiments

**Figure 6:**
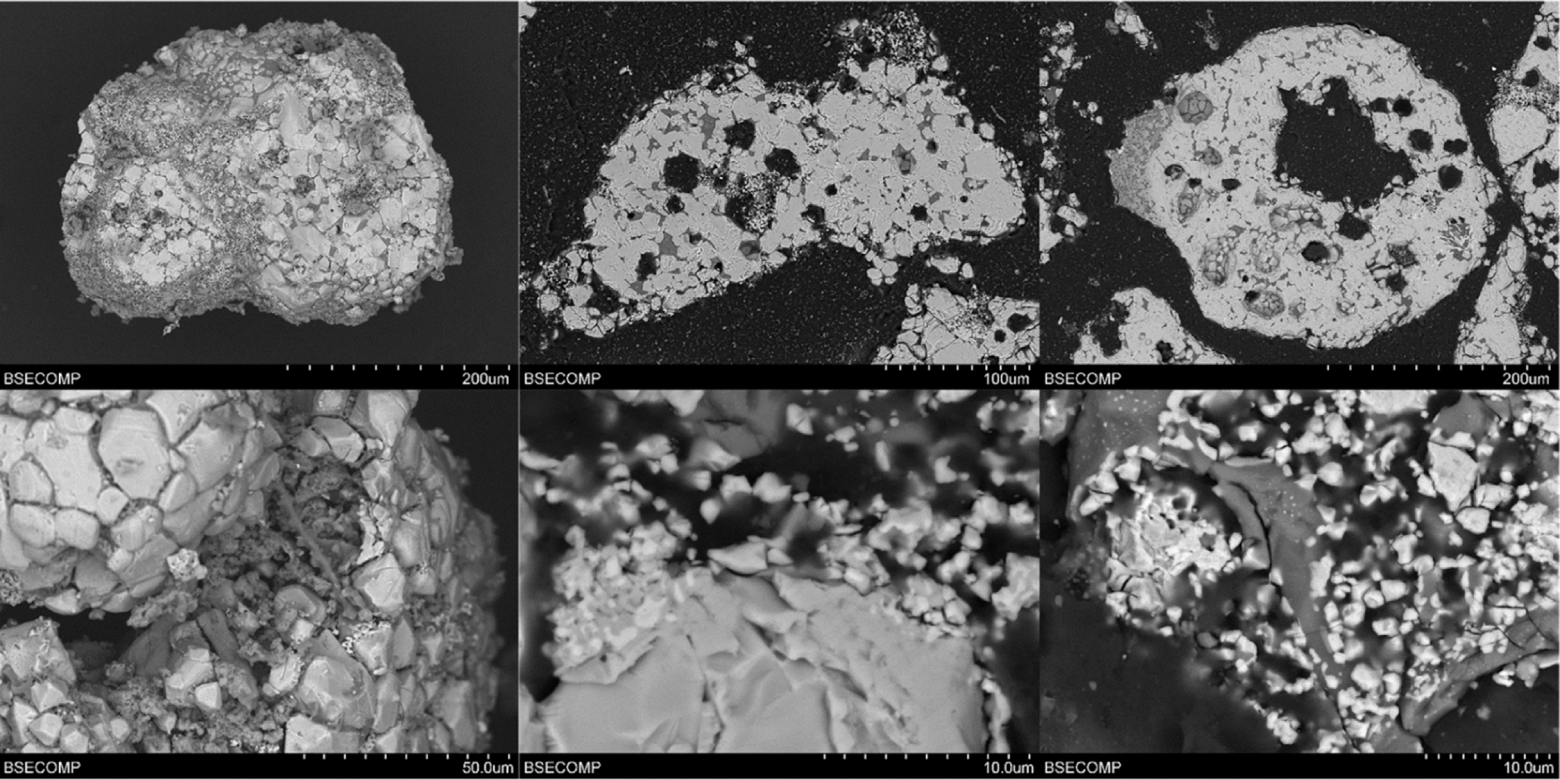
SEM images of Cu30MnFekao7.5 particles after series 1 experiments: (a) Backscattered electrons (BSE) image of a particle; (b) detail of the surface of a particle; (c, e) BSE cross section of a particle; (d, f) detail of the inner part of a particle.

Figure 6 shows the SEM images of the surface (Figure 6a,b) and the cross section (Figure 6c–f) of different Cu30MnFekao7.5 particles. The bright and white sections in the pictures correspond to the Cu–Mn–Fe mixed oxide, and the gray areas correspond to kaolin. It can be seen that no agglomeration between particles was observed. Moreover, no presence was detected of either ash particles on the OC surface or a layer of alkalis on the surface of the particles.

**Figure 7:**
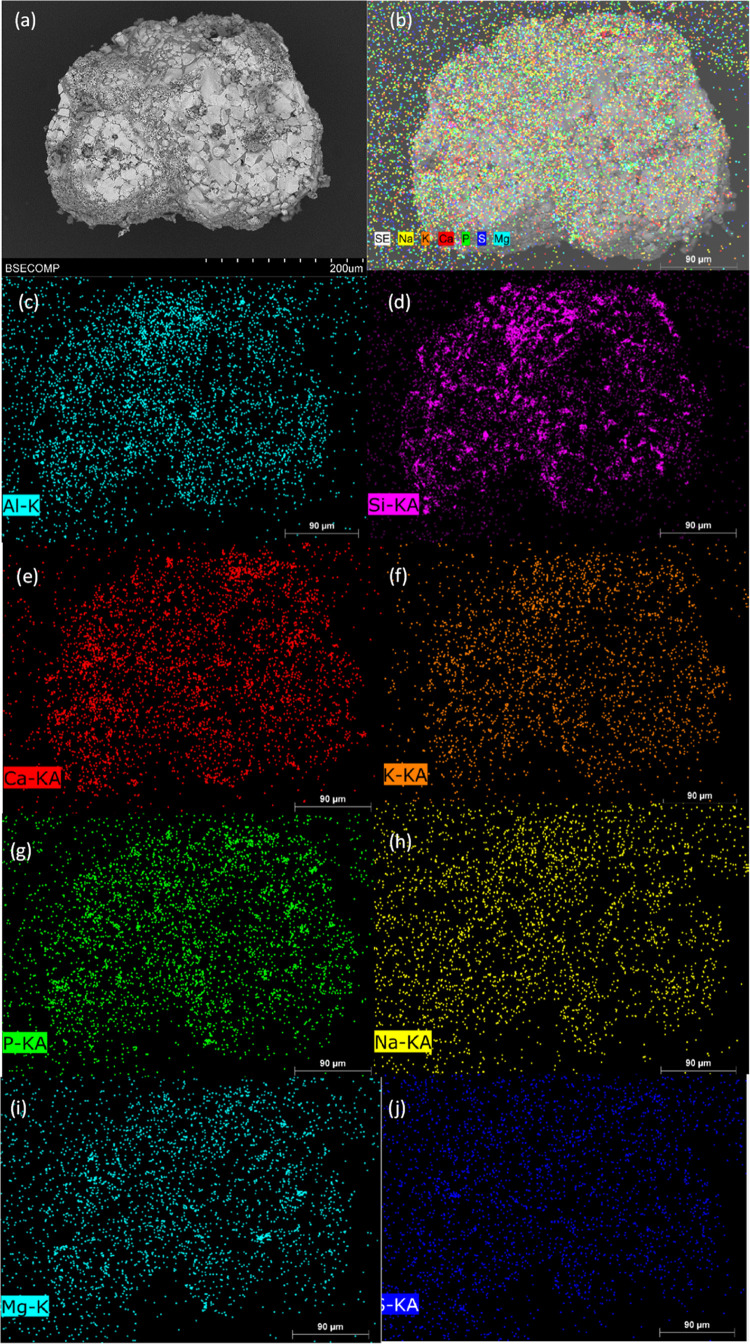
SEM-EDX mapping of different elements present in manure ash on the surface of a Cu30MnFekao7.5 particle after series 1 experiments: (a) SEM image; (b) general mapping; (c) Al-mapping; (d) Si-mapping; (e) Ca-mapping; (f) K-mapping; (g) P-mapping; (h) Na-mapping; (i) Mg-mapping; (j) S-mapping.

EDX mapping of the surface of a Cu30MnFekao7.5 particle was subsequently performed to detect the presence of different elements in the manure ash composition (Figure 7). The general mapping shows a low presence of elements from the ash, and there was no external layer of ash compounds. The ash elements were concentrated in the dark areas, associated with the greater presence of kaolin. With respect to the individual elements, Ca is observed to be predominant, followed by P and K, and there is a lesser presence of Na and Mg, corroborated by the EDX quantification. However, the EDX internal mapping of particles did not find the presence of ash elements diffused to the core.

#### 3.3.2 Effect of Ash-OC Contact Time in CLC/CLOU Experiments

**Figure 8:**
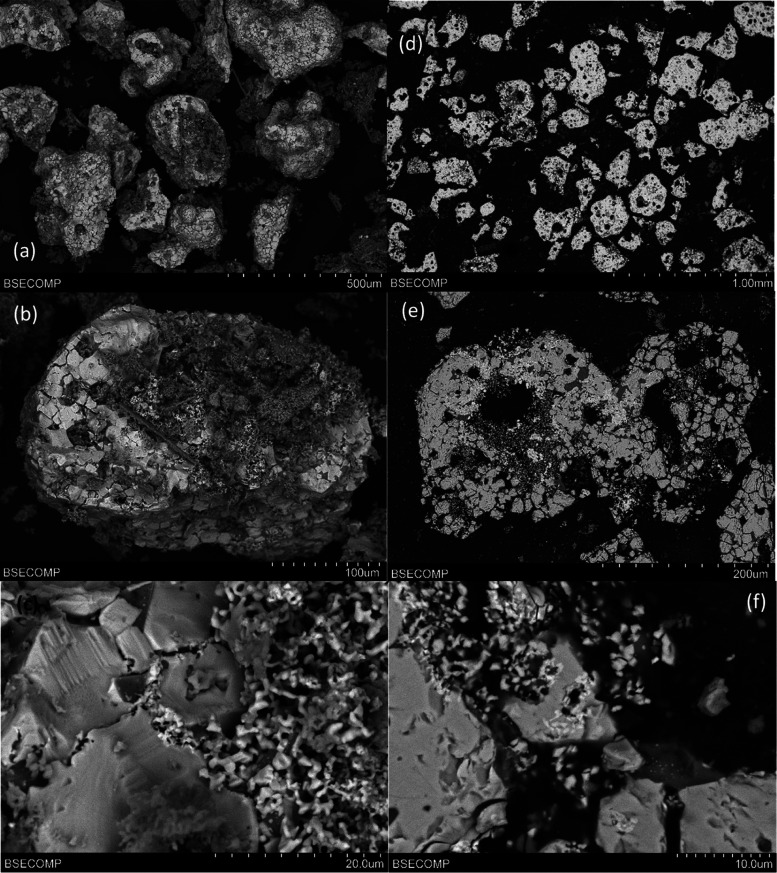
SEM images of Cu30MnFekao7.5 particles after 20 h of series 2 batch experiments: (a, b) BSE image of particles; (c) detail of the surface of a particle; (d, e) BSE cross section of a particle; and (f) detail of the interior of a particle.

With regard to the results obtained for different contact times between the manure ash and OCs, Figure 8 shows SEM images for Cu30MnFekao7.5, where the bright and white sections are Cu–Mn–Fe mixed oxide and the gray areas correspond to kaolin. No agglomeration problems were found. Small amorphous manure ash particles were identified on the surface of the OC particles, but no external layer of manure ash elements (Ca, P, Si, Mg, K, etc.) could be detected over the entire surface of the OC particles in the cross-sectional analysis.

**Figure 9:**
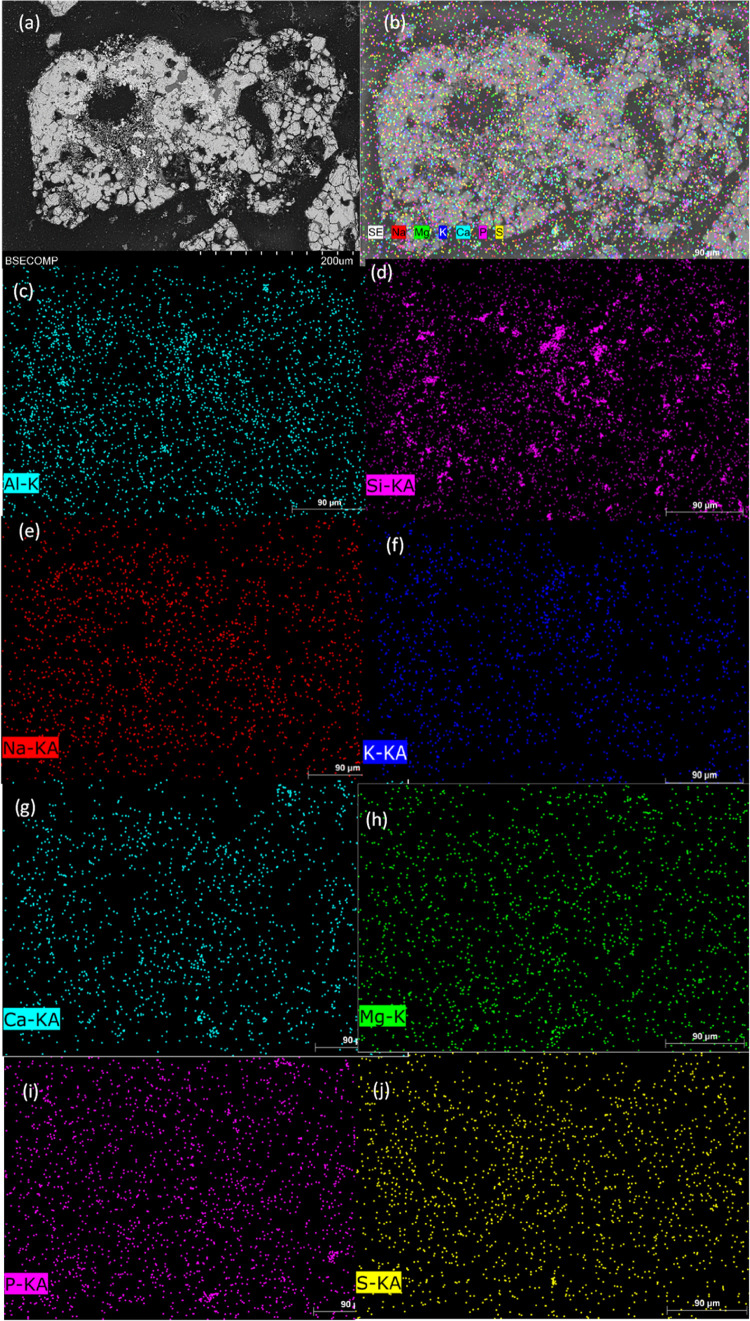
SEM-EDX mapping of different elements present in manure ash inside a Cu30MnFekao7.5 particle after 20 h of series 2 batch experiments: (a) SEM image; (b) general mapping; (c) Al-mapping; (d) Si-mapping; (e) Na-mapping; (f) K-mapping; (g) Ca-mapping; (h) Mg-mapping; (i) P-mapping; (j) S-mapping.

The elements present in manure ash were detected in the ash particles on the surface of the OC particles. Similarly to series 1, they were largely present in kaolin-rich areas. EDX mapping of the cross section of the particles detected K in areas with kaolin-rich areas also inside the particle (see Figure 9d,f). This observation was previously reported by Purnomo et al.,^17^ who found that K can penetrate oxygen carrier particles that are high in Si, such as iron sand (16 wt % Si). Filsouf et al. also observed this behavior during the combustion of pine biomass in a 1.5 kW_th_ CLOU unit with this Cu30MnFekao7.5, where they found that particles were enriched in K, mainly the kaolin-rich areas of the particle interiors.^23^ The presence of the remaining elements was negligible inside the particle because the points detected by the EDX did not correlate with the shape of the analyzed particle (see Figure 9). Therefore, in the presence of kaolin, K can diffuse inside the particle by reacting with kaolin components.

**Figure 10:**
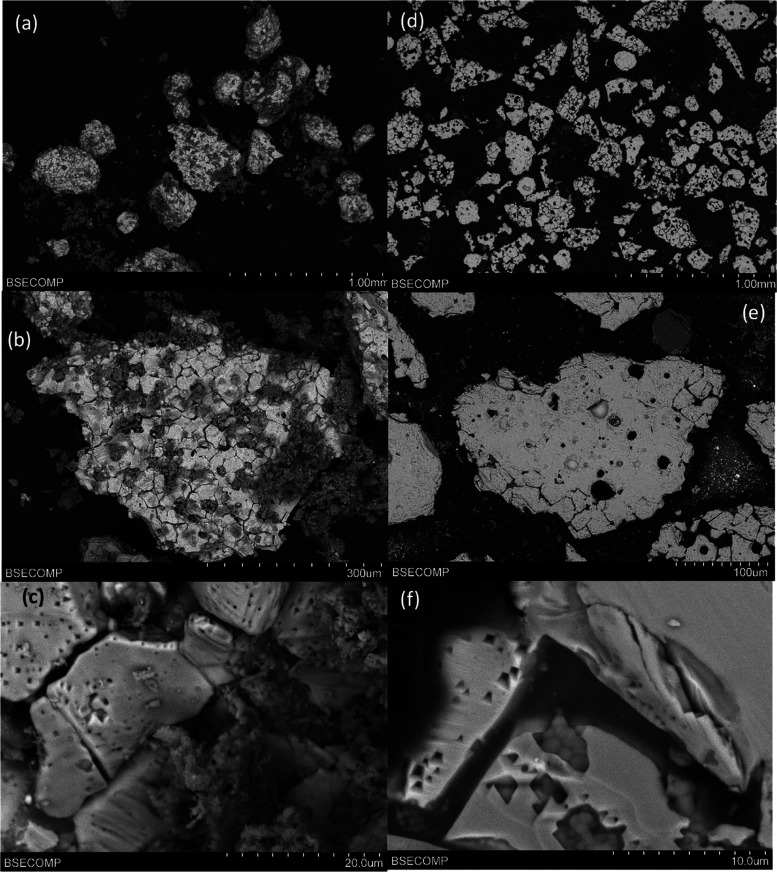
SEM images of Cu30MnFe_Mag particles after 20 h of series 2 batch experiments: (a, b) BSE image of particles; (c) detail of the surface of a particle; (d, e) BSE cross section of a particle; (f) detail of the interior of a particle.

Figure 10 shows SEM images of the surface (Figure 10a–c) and the cross section (Figure 10 d–f) of Cu30MnFe_Mag. The bright and white sections in the images correspond to Cu–Mn–Fe mixed oxide, and the dark gray areas are particles of manure ash over the surface of the oxygen carrier particles. A comparison with the results for Cu30MnFekao7.5 (Figure 8) indicates that there is a lower amount of ash particles on the OC surface (Figure 10a,b). This detail could be associated with the absence of kaolin in the particle. Moreover, the growth of globular crystals enriched in P and Ca was found with this material on the surface of the particle (see Figure 10f). This was not detected for Cu30MnFekao7.5 or ilmenite.

With regard to the presence of elements from manure ash, no external layer of alkalis or phosphorus compounds was detected on the surface of the OC particles, other than the ash particles. EDX mapping performed on the interior of the particles did not show evidence of alkalis or other manure ash compounds migrating inside the particle. Unlike Cu30MnFekao7.5, Cu30MnFe_Mag could be more resistant to the harmful effects caused by the alkalis present in manure ash.

**Figure 11:**
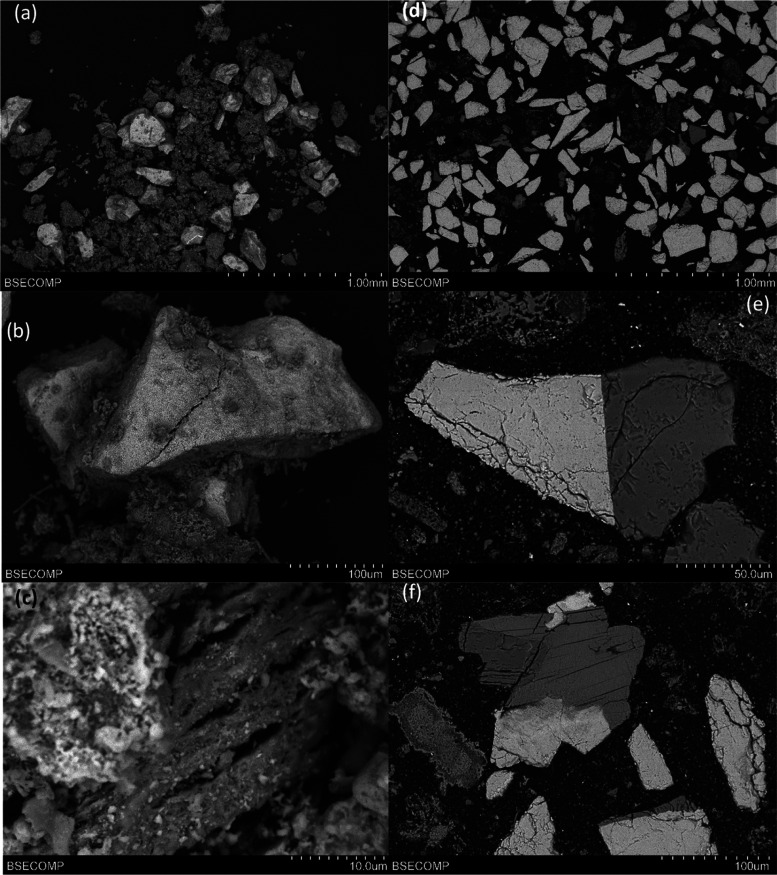
SEM images of Ilmenite particles after 20 h of batch experiments in series 2 experiments: (a, b) BSE image of particles; (c) detail of the bridge between agglomerated particles; (d–f) BSE cross section of a particle.

In the case of ilmenite, which agglomerated in the batch fluidized-bed reactor after 20h of CLC cycles with CH_4_, Figure 11 shows SEM images of the particles after the experiments were carried out. The bright and white sections seen in the images are of ilmenite (Fe–Ti mixed oxide), and the dark gray zones are particles of manure ash over the surface of the ilmenite particles. The images show the agglomeration of the OC particles together with ash particles (Figure 11a,b,d). The structure of the ash bridge between two ilmenite particles can be observed in Figure 11c. This structure was not detected with CLOU OCs, and this interaction is considered to be the cause of the agglomeration. EDX analysis of the bridges found that they are mainly composed of P, Mg, K, and Ca.

**Figure 12:**
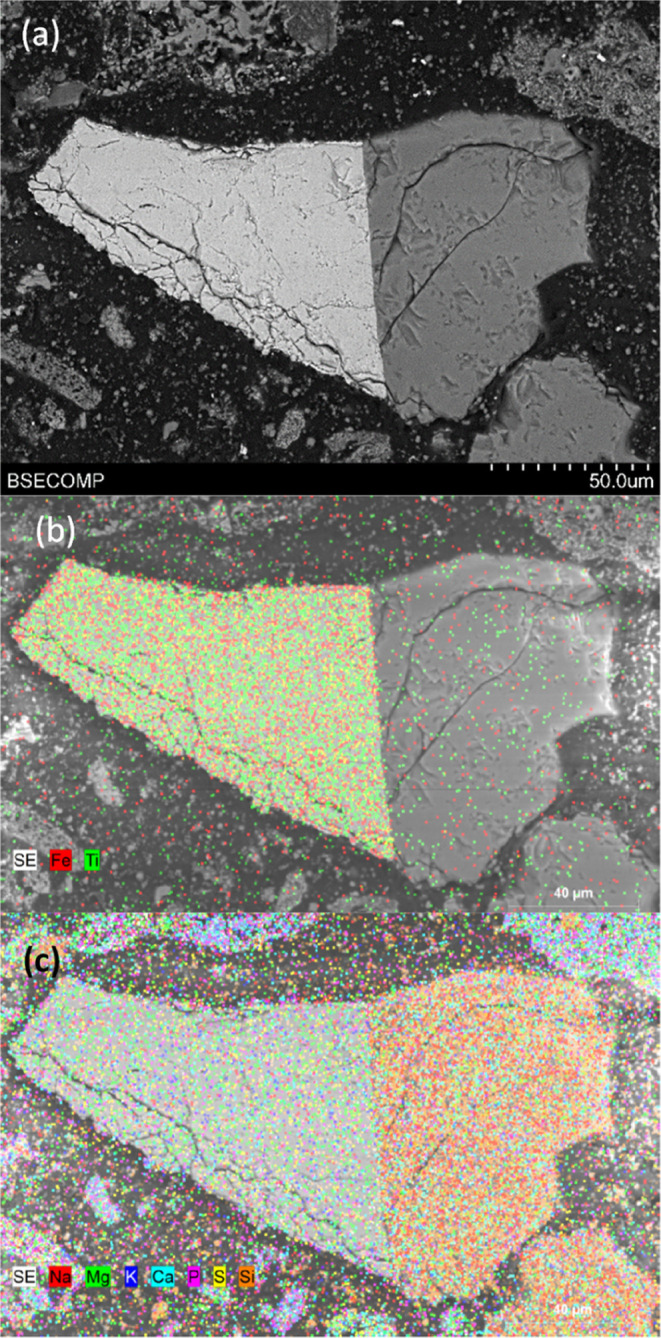
SEM-EDX mapping of different elements present in manure ash inside an ilmenite particle after series 2 experiments: (a) SEM image; (b) Fe–Ti-mapping; (c) Na–Mg–K–Ca–P-S-Si-mapping.

Moreover, Figure 11e,11f show structures in which two particles, one of ilmenite and the other of ash, are perfectly bonded; it can be said that these particles are welded. The EDX analysis of the particle in Figure 11e can be found in Figure 12. In this case, the left side of the particle consists of ilmenite (Figure 12b) and the right side is a manure ash particle (Figure 12c), with a very clear boundary between both sides. The ash particles in this case are mainly composed of Na, Ca, and Si. In addition, Mg can be found on the ilmenite side of the particle. This behavior was found in many of the particles analyzed by SEM-EDX. The Mg can be associated with impurities in the ilmenite ore, in the same way that kaolin was previously found as an impurity in ilmenite.^37^

## 4 Conclusions

This study analyzed the interactions of three different OCs (two synthetic magnetic Cu-based CLOU OCs and ilmenite) with swine manure ash. CLOU and CLC redox cycles were run in a batch fluidized-bed reactor. Both CLOU OCs did not show agglomeration problems after 20 h of CLOU and CLC cycles. For CLOU OCs, the concentration of released oxygen depended on OC conversion. The ash fraction present did not significantly affect the concentration of released O_2_ for ash fractions lower than 16 wt %.

After CH_4_ combustion in a CLOU process, the OC behavior showed activation, particularly that of OC containing kaolin (Cu30MnFekao7.5). During CH_4_ combustion with ilmenite, CH_4_ conversion was incomplete, and the OC experienced hard agglomeration after 20 h of CLC cycles, indicating that ilmenite is not a suitable OC for the CLC of swine manure.

With regard to the interaction between the OCs and the ash compounds, for the longer interaction periods of all of the oxygen carriers with manure ash, ash particles were found all over the OC surface, with their presence being more accentuated over Cu30MnFekao7.5 and ilmenite. The common point between both is the presence of minerals, such as kaolin in Cu30MnFekao7.5, and other mineral impurities in their composition. Therefore, the presence of minerals in the OCs, either as a support or as impurities, could be associated with greater interaction with ash. Moreover, in the case of Cu30MnFekao7.5, there was diffusion of K inside the OC particles, mainly into kaolin-rich areas.

Finally, the high concentration of swine manure in the bed may have affected the O_2_ release of the CLOU oxygen carriers. However, neither of these OCs exhibited agglomeration problems or a decrease in their reactivity. Furthermore, as these CLOU OCs have magnetic properties that improve their separation from fuel ash, they make good candidates for CLC using swine manure as a fuel by controlling the effect of the ash in the process.
